# Evaluation of the Effectiveness of a Psychoeducational Intervention in Treatment-Naïve Patients with Antidepressant Medication in Primary Care: A Randomized Controlled Trial

**DOI:** 10.1155/2015/718607

**Published:** 2015-08-25

**Authors:** R. Casañas, R. Catalán, R. Penadés, J. Real, S. Valero, MA. Muñoz, LL. Lalucat-Jo, M. Casas

**Affiliations:** ^1^Research Department, Centre Higiene Mental (CHM) Les Corts, C/Numancia, 103-105 Bajos, 08029 Barcelona, Spain; ^2^Psychiatry and Legal Medicine Department, Universidad Autónoma de Barcelona, Barcelona, Spain; ^3^Primary Healthcare University Research Institute IDIAP-Jordi Gol, Institut Català de la Salut (ICS), C/Sardenya 375, 08036 Barcelona, Spain; ^4^Clinical Institute of Neurosciences (ICN), Hospital Clinic, Barcelona, Spain; ^5^Department of Psychiatry and Clinical Psychobiology, University of Barcelona, Barcelona, Spain; ^6^Institut d'Investigacions Biomèdiques August Pi i Sunyer (IDIBAPS), Barcelona, Spain; ^7^Centro de Investigación Biomédica en Red de Salud Mental (CIBERSAM), Madrid, Spain; ^8^Department of Psychiatry, Hospital Universitari Vall d'Hebron, Universidad Autónoma de Barcelona (UAB), Barcelona, Spain

## Abstract

*Background*. There is evidence supporting the effectiveness of psychoeducation (PE) in patients with symptoms of depression in primary care (PC), but very few studies have assessed this intervention in antidepressant-naïve patients. The aim of this study is to assess the effectiveness of a PE program in these patients, since the use of antidepressant (AD) medication may interfere with the effects of the intervention. *Methods*. 106 participants were included, 50 from the PE program (12 weekly 1.5-hour sessions) and 56 from the control group (CG) that received the usual care. Patients were assessed at baseline and at 3, 6, and 9 months. The main outcome measures were the Beck Depression Inventory (BDI) and remission based on the BDI. The analysis was carried out on an intention-to-treat basis. *Results*. The PE program group showed remission of symptoms of 40% (*P* = 0.001) posttreatment and 42% (*P* = 0.012) at 6 months. The analysis only showed significant differences in the BDI score posttreatment (*P* = 0.008; effect size Cohen's *d*′ = 0.55). *Conclusions*. The PE intervention is an effective treatment in the depressive population not treated with AD medication. Before taking an AD, psychoeducational intervention should be considered.

## 1. Introduction

Depression is one of the most prevalent mental disorders in the adult population worldwide, with a lifetime prevalence of 9–20% [1] and specifically 10.5% in Spain [2].

In primary health care there has been an increase in the detection of major depressive disorders in recent years, with a 12-month prevalence of 11% in Europe and 14% in Spain [3]. In fact, minor depressive disorders are the third leading cause of consultation in primary health care, with a prevalence rate of 5–16% [4–6], and it is an important risk factor for major depression, which develops in 10–25% of patients with subthreshold depression within 1–3 years [7].

A significant increase in the prescription of antidepressants in primary care in recent decades in Spain has been confirmed [8], possibly due to the use of so-called “third-generation” antidepressants, as selective serotonin reuptake inhibitors (SSRIs) are the chosen treatment among patients with depression.

When we reviewed the interventions that have proven effective in treating minor depression, we found that most international clinical practice guidelines (CPG) for the management of depression recommend psychoeducational interventions and brief psychotherapy as an initial step in the treatment protocol [9–11]. These guidelines do not recommend antidepressant medication in patients with mild symptoms.

With regard to psychoeducation, it has been demonstrated that it is an effective therapy in the treatment of depression in adults [9, 12], as it reduces depressive symptoms and can prevent depression in primary care patients [13–15]. It has also been proven to reduce depressive symptoms in mild and moderate depression in both the short term and long term [16–19].

This intervention could be carried out by community nurses with previous training in primary care [18–22].

In most of these studies, we detected that the antidepressant medication variable had not been taken into account when evaluating whether it might have affected the results of the intervention. Some studies explain that some patients were taking antidepressants [19, 20], while in other studies this variable is omitted [23], even though the few studies that analyzed whether taking antidepressants might have an effect on the effectiveness of the intervention [17] have proven that the results obtained were maintained despite excluding participants taking AD medication.

We carry out a randomized, controlled, open-label, parallel-group trial [18] to assess the effectiveness of a psychoeducational program versus the usual care in a sample of 231 patients diagnosed with major depression (mild/moderate symptoms) recruited at 12 urban primary care centers (PCCs) in Barcelona. The intervention group (*n* = 119) participated in a psychoeducational program (12 weekly 1.5-hour sessions led by two nurses) and the control group (*n* = 112) received the usual care. This group program included aspects of personal care and a healthy lifestyle (diet, physical exercise, sleep, and pharmacological treatment), as well as the identification and management of depressive symptoms within the psychoeducational intervention and cognitive-behavioral techniques used in psychoeducation.

The results of the study showed that this psychoeducational intervention was more effective in patients with mild symptoms, since they had a higher symptom remission rate over the short terms and long term. Moreover, this improvement was associated with better quality of life. The data do not demonstrate that the intervention is effective over the long term in patients with moderate symptoms [18].

In this paper, the main objective is to assess the effectiveness of this intervention through the rate of remission in the sample of antidepressant-naïve patients. Among the secondary objectives, we were interested in analyzing how many patients had taken ADs during the intervention and at 6 and 9 months of follow-up, whether taking medication was associated with worsening of symptoms and whether the number of group sessions attended influenced improvement in symptoms.

## 2. Methods

A detailed description of the methodology has been reported previously [18]. In this study we will only specify the most important methodological aspects. The randomized, controlledtrial was conducted between December 2008 and April 2010 in Barcelona, Spain. Participants were recruited by general practitioners and nurses between December 2008 and March 2009 at 12 PCCs.

### 2.1. Participants

231 participants were included in the study [18] and randomly assigned to the intervention group (IG) (*n* = 119) or the control group (CG) (*n* = 112). The subgroup of patients who had never taken pharmacological antidepressant treatment prior to participating in the study (*n* = 106) was extracted from this patient sample.

Inclusion criteria were (a) patients included in the study [18] who had never been treated with antidepressant medication; (b) male and female patients over 20 years of age; (c) patients diagnosed with a major depressive disorder according to the International Classification of Diseases 10th revision (ICD-10) [24]; (d) patients with mild to moderate symptoms according to the Beck Depression Inventory (BDI ≥ 10 and < 30); and (e) provision of signed informed consent.

Exclusion criteria were as follows: (a) patients who had been treated with ADs some time prior to participating in the study.

The information about antidepressant prescription was obtained from the primary care information system.

### 2.2. Procedure

Of the 231 patients included in the main study, 106 were included in this study.

All outcome variables were assessed four times: prior to start of the study (pretest), after 3 months (posttest), and at 6 and 9 months after inclusion (first follow-up and second follow-up, resp.) in individual data collection sessions.

### 2.3. Measures

Participant diagnosis was based on the International Classification of Diseases 10th revision (ICD-10) [24]. The diagnosis was made by the general practitioner (GP).

#### 2.3.1. Beck Depression Inventory

The Beck Depression Inventory is a brief scale of 21 items which assesses the severity of depressive symptoms during the previous week. The score ranges from 0 to 63 points. The usually accepted cut-off points for classifying the intensity/severity are as follows: no depression: 0–9 points, mild depression: 10–18 points, moderate depression: 19–29 points, and severe depression: ≥30 points [25].

#### 2.3.2. Remission

Clinical remission is based on the BDI, which is a self-reporting screening instrument. Remission is defined as a mean BDI score of ≤11 [26]. On the BDI self-rating scale, a cut-off of BDI ≤ 11 emerged for remission with a sensitivity of 90% and specificity of 64%.

### 2.4. Group Treatments

#### 2.4.1. Description of the Psychoeducational Group Intervention

The intervention consisted of 12 weekly 90-minute sessions led by two nurses. Each group consisted of 8–12 participants.

The program provided (1) health education about the illness: symptoms, diet, physical exercise, sleep, pharmacological treatment, and adherence to treatment; (2) breathing techniques; (3) problem solving, behavioral activation, and a cognitive-behavioral approach to depression; (4) self-esteem and self-image; and (5) pleasant activities, social skills, and assertiveness [27].

To enhance the active role of the patient, each session was accompanied with homework for the patient.

#### 2.4.2. Description of the Control Group

Patients from the control group were no longer taking AD medication. Members of the control group received the usual treatment (visits with GPs and nurses). There was no pattern of visits established; the patients could go to the PCC when they needed to. The GPs and nurses used their own criteria to care for depressed patients.

### 2.5. Analysis

The analysis was carried out on an intention-to-treat basis. The analyses were based on the data of the 106 participants who completed some of the evaluations. The intention-to-treat analysis was carried out as follows: missing values were replaced by the scores from the previous assessment (the last observation carried forward (LOCF)) to ensure no increase. To examine baseline differences in the sociodemographic and clinical characteristics between groups, Student's *t*-test was applied for continuous variables and the Chi-square test for categorical variables.

The effect of the intervention on the outcome variables was measured by means of the difference in scores between groups and the effect size. Standardized effect size (SES) [28] is calculated as the mean difference between the intervention and the control groups, divided by the standard deviation (SD) of the control group. The SES is a standardized measure of the change that enables comparison between groups, between measures in the same study, and between different studies [29].

The standardized response mean (SRM) was used to measure the effect size within group comparisons. The SRM was calculated as the mean change divided by the SD of the change. Cohen's *d* allows the effect size to be classified into small (0.2 to 0.5), medium (0.5 to 0.8), and large (0.8 or over); these criteria can also be applied to the SRM [29, 30]. The IBM SPSS Statistics v.18 statistics package was used [31].

To evaluate the evolution of BDI scores between groups, we performed repeated measures of analysis of variance (ANOVA). We evaluated the goodness of fit using the Kolmogorov-Smirnov test of the residuals.

To evaluate the possible relationship between the number of sessions and the decrease in the BDI score in the intervention group, the Spearman correlation coefficient (*r*) was calculated for each time.

## 3. Results

**Figure 1:**
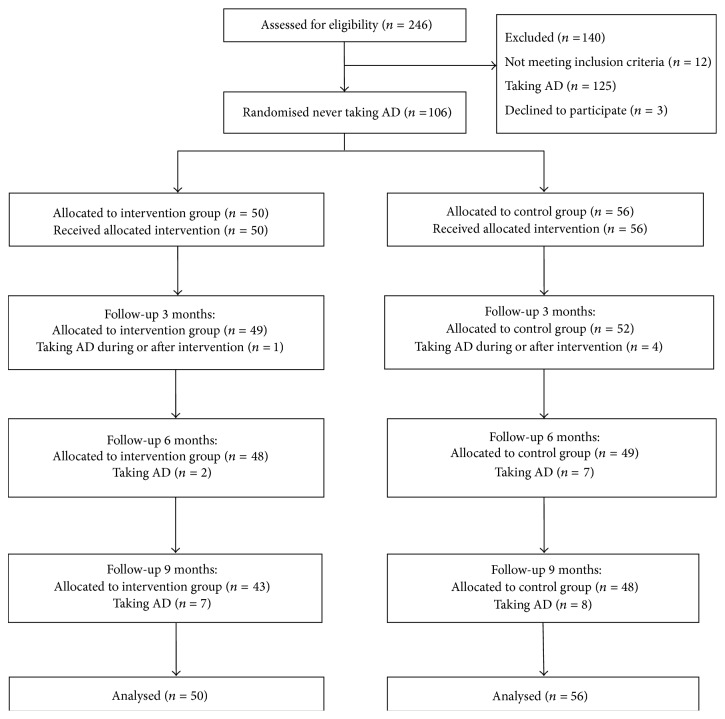
Flow chart of participants.

The flow of participants is shown in Figure 1. Of the 246 allocated to the study, 140 were excluded: 125 were or had been on AD treatment, 12 did not meet the inclusion criteria, and 3 people chose not to participate.

### 3.1. Patient Characteristics

106 patients were included in the study, 50 corresponding to the PE Group and 56 to the control group. These two groups were similar at baseline in terms of demographic and clinical characteristics, except with respect to gender (*P* = 0.018) and hypnotic medication (*P* = 0.028).  Table 1 shows the baseline characteristics of the total study population and the intervention and control group. The typical patient was a native Spanish woman, approximately 53 years old, married/cohabiting, with primary studies, and self-employed. She had zero or two children and referred to a stressful event in the previous month.

Those allocated to the psychoeducational group received a mean of 8.74 (SD 4.26; range 1–12) sessions. Adherence to psychoeducational intervention was reasonably good, 38 (76%) receiving at least eight sessions or more. The sessions received by the intervention group were 12 sessions (*n* = 21); 11 sessions (*n* = 4); 10 sessions (*n* = 8); 9 sessions (*n* = 3); 8 sessions (*n* = 2); 3 sessions (*n* = 1); 2 sessions (*n* = 4); and 1 session (*n* = 7).

### 3.2. Attrition and Dropout

Of the sample of 106 patients included in the study, 26 were dropouts (dropouts = patients who were not evaluated at posttreatment and follow-up assessments at 6 and 9 months). Therefore, the overall dropout rate was 24.52%. The dropout rate was 20% (*n* = 10) in the intervention group and 28.57% (*n* = 16) in the control group. Dropouts from the experimental group did not differ statistically from those in the control group at follow-up assessments. The overall dropout rate was 23% of the initial study [18].

### 3.3. Intervention Effectiveness: Remission

The proportion of patients achieving remission (BDI score of ≤11) was examined using the Riedel remission criteria for major depression [26].

Posttest results showed that more participants in the intervention group (*n* = 20) had scored in the nonsymptomatic BDI range (BDI score of ≤11) than participants in the control group (*n* = 7). This means that 40% of the participants in the intervention group and 12.5% in the control group did not have depressive symptoms; the 27.5% difference between groups was statistically significant (*P* = 0.001, 95% CI 11.4 to 43.6). After 6 months of follow-up the results were similar: the proportion was 42% in the intervention group and 19.6% in the control group; the 22.4% difference between groups remained statistically significant (*P* = 0.012, 95% CI 5.2 to 39.6). After 9 months of follow-up, the proportion was 44% in the intervention group and 26.8% in the control group; however, the 17.2% difference between groups was not statistically significant (*P* = 0.064, 95% CI −35.5 to 0.79).

Table 2 shows the proportion of patients in the overall sample remitting through treatment.

The number needed to treat (NNT) to achieve remission is about 4 at 3 months (CI 95% 2.3 to 8.8), 5 at 6 months (CI 95% 2.5 to 19.3), and 6 for the long-term (after 9 months), that is, reducing the BDI score below 11.

### 3.4. Depressive Symptoms

Depressive symptoms were assessed using the Beck Depression Inventory (BDI). The difference between treatments at 3 months (psychoeducational intervention minus control) was estimated to be −3.61 (95% CI −6.25 to −0.95), which was significant (*P* = 0.008). The negative sign indicates that participants in the psychoeducational intervention group showed a greater decrease in depressive symptoms than those in the control group. The results at 6 and 9 months were not significant.  Table 3 shows the changes in BDI score within and between the intervention and usual care groups, with missing data replaced using the last value carried forward.

**Figure 2:**
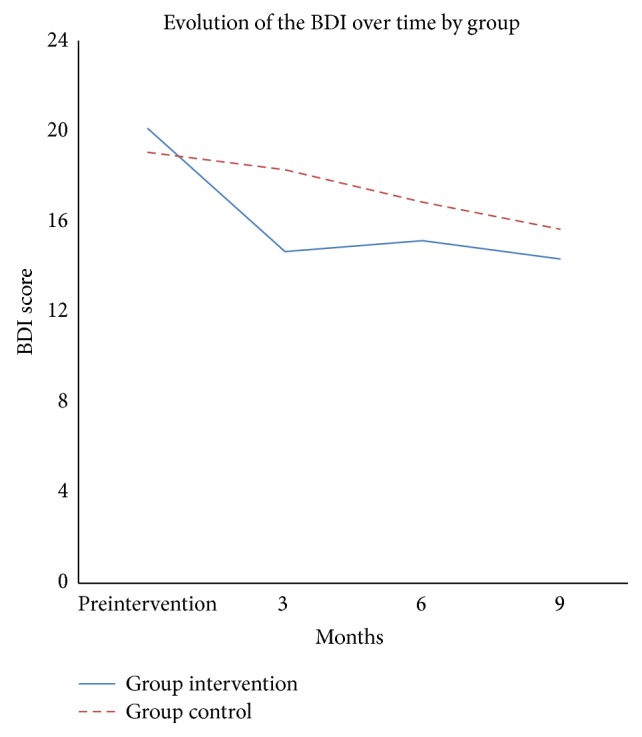
Evolution of the BDI score over time by group.

The results showed that the evolution of the BDI scores over time between groups was significant in a nonlinear trend (*P* value nonlinear trend = 0.001). The effect size of this contrast was moderate (*d*′ = 0.55) in the short term (posttest) and smaller (*d*′ = 0.18) in the long term (at 9 months of follow-up).  Figure 2 shows the evolution of the BDI score over time by groups.

As a secondary analysis, we were also interested in analyzing the evolution of the BDI score over time in the intervention group and control group separately.

Within the intervention group, a reduction was observed in the BDI score of 5.50 and 5.80 points posttreatment and at 9 months of follow-up, respectively. In contrast, in the control group this reduction was only 0.80 and 3.40 points posttreatment and at 9 months of follow-up, respectively (Table 3).

By analyzing the intervention and control groups separately, the effect size within the intervention group (SRM) was high over time (*d*′ = 0.80 postintervention and *d*′ = 0.75 at 9 months of follow-up) and the effect size within the control group was small over time (*d*′ = 0.16 postintervention and *d*′ = 0.44 at 9 months of follow-up) (Table 3).

### 3.5. Antidepressant Treatment

Out of the 106 patients included in the study, 16 patients started AD treatment during the study, 7 from the IG and 8 from the CG. If we specify the time when AD treatment was prescribed, we find that 5 people were taking ADs after intervention, IG (*n* = 1) and CG (*n* = 4); at 6 months of follow-up (*n* = 9), IG (*n* = 2), and CG (*n* = 7); and at 9 months of follow-up (*n* = 15), IG (*n* = 7), and CG (*n* = 8). It should be mentioned that out of all the patients who initiated AD treatment during the study, only one patient from the CG initiated AD treatment after intervention and stopped after a month. The remaining patients continued with the prescribed treatment throughout the follow-up.

### 3.6. Number of Sessions and BDI

Regarding the number of sessions attended, an inverse relationship has been observed between the fall in BDI score compared to baseline and the number of sessions attended. Thus, participants who attended more sessions had a greater decrease in their BDI score both at 3 months (*r* = −0.354, *P* value = 0.012) and at 9 months of follow-up (*r* = −0.333, *P* value = 0.018).

## 4. Discussion

We found a relationship between the psychoeducational group intervention and the remission of depressive symptoms in this sample of patients not taking antidepressants.

More patients from the IG had remission of their depressive symptoms (BDI score ≤ 11) [26] in the short term (posttreatment) and long term (at 6 and 9 months of follow-up) compared with the control group. The psychoeducational group intervention proved to be effective in the short term, showing a reduction of 5 points in the BDI score and this symptomatic improvement in BDI continued to follow-up at 9 months. In contrast, the control group needed 9 months to achieve a 3-point improvement in BDI. We could say that it is an effective intervention over the short term, although the effect size is moderate (effect size Cohen's *d*′ = 0.55).

In this study, we do not analyze what type of population may benefit most from receiving the group intervention, whether participants are with mild (BDI score ≤ 11) or moderate (BDI score ≤ 18) depression. Of the 106 participants included, 47 had mild and 59 had moderate depression symptoms; therefore, the samples were too small for significant conclusions to be drawn.

Our results show that after 3 months (after intervention or short term), a considerable improvement is achieved in terms of both symptoms and symptom remission. It could be said that the psychoeducational group intervention reduces the duration of depressive episodes after a 3-month period, since the results show that 40% of the patients in the intervention group did not have postintervention depressive symptoms, compared to 12.5% in the control group, and this difference of 27.5% between groups was significant (*P* = 0.001). At 6 months of follow-up, the results remained constant (*P* = 0.012); however, after 9 months, the difference of 17.2% between groups was not significant (*P* = 0.064).

The data were consistent with those found in the study by Allart-van Dam et al. [16], which showed that 52.5% of the intervention group did not present postintervention depressive symptoms (BDI < 10) [25] versus 31.7% in the control group, with a significant difference of 20.8% (*P* = 0.04) between groups.

When we talk about the remission of symptoms in terms of number needed to treat (NNT), we observed that our NNT of 4 postintervention, 5 at 6 months, and 6 at 9 months of follow-up are supported by those obtained in the study by Dalgard [19] with a smaller sample of patients (*n* = 155), which was 6 at 6 months of follow-up, and the ODIN study [20] (*n* = 452), which was 7 at 6 months, supporting the effectiveness of the intervention. It must be mentioned that in these two studies the patients were taking ADs, but they did not analyze whether the pharmacological treatment could interfere in the results of the intervention.

Our results show a significant improvement in symptoms after intervention (*P* = 0.008; *d*′ = 0.55), although the effect size is moderate, but this improvement is not maintained at 9 months of follow-up (*P* = 0.39; *d*′ = 0.18). Our results coincide with those found in a review [32] which showed that psychological treatment for minor depression, including psychoeducation, is effective over the short term (*d*′ = 0.42) and studies that had already demonstrated the effectiveness of psychoeducation over the short term [16, 22, 23]. With respect to the duration of the therapeutic effect of psychoeducation at 6 and 12 months of follow-up, the results are controversial.

These data also match those found in the study by Allart-van Dam et al. [17] (*n* = 104), which showed that the effect of the psychoeducational intervention was maintained after 6 and 12 months, even excluding participants who had taken ADs during the intervention (*n* = 18).

Based on the observation of results, it could also be stated that psychoeducational group intervention delays the prescription of ADs. ADs were prescribed to more patients from the CG during the intervention and at 6 months and 9 months of follow-up as compared to the IG.

If the patients from the IG that received ADs during the study (*n* = 7) are analyzed in more detail, we find that the only patient that received AD treatment after intervention and carried on taking it during the follow-up had only attended 2 group sessions and was only evaluated at baseline, with a BDI score of 27. The rest of the patients to whom an AD had been prescribed during the 9-month follow-up had attended an average of 11 sessions and their BDI score had decreased or was stable during follow-up, being 18.50 (SD = 6.60) at 9 months. None of their BDI scores was higher than 30. In the control group, more patients received postintervention AD treatment (*n* = 4) during the 6- and 9-month follow-up (*n* = 7 and *n* = 8, resp.), and two of them had a BDI score of 32 and 37 at postintervention time.

Good adherence to the psychoeducational therapy was observed, with 42% (*n* = 21) of the participants attending all 12 sessions and 76% (*n* = 38) attending at least 8 sessions. 24% (*n* = 12) of the patients showed poor adherence to treatment, as they only attended between 1 and 3 sessions. Although there are few studies that have evaluated adherence to psychological therapies in patients with depression [33, 34], a meta-analysis aimed at identifying effectiveness predictors in depression preventive programs [35] reached the conclusion that programs of more than 8 sessions with a 60–90 minute duration offered a large effect size and that the number of sessions is important for the patient to internalize the knowledge, the processes, and the skills learned during the intervention, meaning that fewer than 8 sessions are likely to be insufficient.

However, there are quite a lot of studies that have analyzed adherence to pharmacological treatment. Their conclusion is that there is low adherence to antidepressant treatment in primary care [36–38], most patients do not follow treatment recommendations [37, 38], low concordance is observed between the real practice of primary care physicians and CPG recommendations regarding depression [39], and a systematic review does not recommend antidepressants for the initial treatment of subjects with minor depression [6].

Psychoeducation has proven effective as psychotherapy for depressive symptom management in the primary care setting [16, 17, 19, 20, 22, 23], and it can be carried out by community nurses [19–22]. A systematic review about the effectiveness of psychoeducation for depression [40] suggests that psychoeducation is effective in improving the clinical course, treatment adherence, and psychosocial functioning of depressive patients.

Even though no consensus has been achieved on the definition of psychoeducation, all psychoeducational interventions share a group structure, some homework, and an educational approach [18, 35], even if they use different methods (cognitive-behavioral therapy [41], coping with depression course (CWD) based on cognitive-behavioral techniques [17, 19, 20], or multicomponent interventions [22]).

In general, psychotherapeutic and psychosocial interventions for depression in primary care are aimed at improving compliance with therapy and offering a therapeutic alternative to drugs. Some studies suggest the importance of having effective treatments for depression in primary care [42, 43] or increasing access for patients with depressive symptoms in primary care to psychological therapies that have proven effective in the short run, as they can have more prolonged benefits [44]. Studies show that the community could be a suitable setting in which to send patients for preventive interventions in cases where the person has a high symptom score but does not meet the criteria for major depression [42].

According to the results, this PE intervention is effective in the short term, with high rates of remission in patients with mild and moderate depressive symptoms not treated with AD medication. These results coincide with those found in an earlier study [18] which showed that this psychoeducational intervention was more effective in patients with mild symptoms, since they had a higher symptom remission rate over the short term and long term, but the intervention was not shown to be effective over the long term in patients with moderate symptoms.

Due to the high prevalence of depression in primary care and the increase in spending on antidepressants, it is necessary to implement interventions that have proven to be effective and that could contribute to an improvement in depression management and reduce its high costs.

*Strengths and Limitations of This Study*. Our trial has several strengths: firstly, it is the first study to assess the effectiveness of this psychoeducational group intervention in patients not treated with AD medication. The intervention includes health education about the disorder, healthy behaviors, social skills, and cognitive-behavioral techniques. Secondly, this study is the first multicenter, randomized study that assesses the effectiveness of a psychoeducational intervention in Spain. Thirdly, the sample population was representative of the whole of Barcelona. The participating PCCs were located in various areas throughout Barcelona, with different sociodemographic and economic resources.

Despite the positive findings, potential biases need to be considered when evaluating the study. Some of the limitations of the study could be as follows: firstly, we performed a randomization of patients, but with no double-blind the patients know who belongs to the intervention group and who belongs to the control group, as do the nurses and doctors at the PCC. It was difficult for researchers to remain blinded to group allocation. However, participants completed mood self-rating assessments. Therefore, that lack of blindness should not have affected our primary outcome to a great extent.

Secondly, the sample size is small, containing only 106 patients. Thirdly, the study employed only one outcome measure, BDI, as we wanted the study to be as close as possible to the usual practice at primary care centers. It is a naturalistic study. Thirdly, the remission of depression was assessed by a screening questionnaire (BDI) rather than a diagnostic interview. Fourth, the overall dropout rate was 24.52%. Dropouts from the experimental group did not differ statistically from those in the control group at follow-up assessment. The overall dropout rate was 23% of the initial study [18]. Further studies are required to confirm these results.

## 5. Conclusions

Our results show that this psychoeducational group intervention could be an effective treatment in the population with mild/moderate depressive symptoms not treated with antidepressant medication in primary care over the short term. Before taking an AD, psychoeducational intervention should be considered.

## Figures and Tables

**Table 1:** Baseline characteristics of the total study population and the intervention group. Values are expressed as numbers (percentages).

Variable	Category	(*n* = 106) general *n* (%)	(*n* = 50) intervention *n* (%)	(*n* = 56) control *n* (%)
Gender^*^	Women	96 (90.6)	49 (98)	47 (83.9)

Age	Mean (SD)	52.79 (13.98)	52.14 (13.22)	53.38 (14.71)

Nationality	Spanish	97 (91.5)	45 (90)	52 (92.9)

Marital status	Single	18 (17.1)	9 (18)	9 (16.4)
	Married/cohabitant	51 (48.6)	25 (50)	26 (47.3)
	Divorced/separated	15 (14.3)	8 (16)	7 (12.7)
	Widow/widowed	21 (20)	8 (16)	13 (23.6)

Educational level	Did not complete primary education	13 (12.5)	6 (12)	7 (13)
	Completed primary education	38 (36.5)	18 (36)	20 (37)
	Secondary education	33 (31.7)	18 (36)	15 (27.8)
	University	20 (19.2)	8 (16)	12 (22.2)

Number of children	0 children	31 (29.2)	12 (24)	19 (33.9)
	1-2 children	51 (48.1)	25 (50)	26 (46.4)
	≥3 children	24 (22.6)	13 (26)	11 (19.6)

Employment status	Self-employed	97 (42.4)	56 (47.1)	41 (37.3)
	Disability or permanent disability	20 (8.7)	9 (7.6)	11 (10)
	Unemployed	32 (14)	18 (15.1)	14 (12.7)
	Works at home	36 (15.7)	19 (16)	17 (15.5)
	Retired	44 (19.2)	17 (14.3)	27 (24.5)

Core coexistence	Alone	22 (21.2)	8 (16)	14 (25.9)
	With children	15 (14.4)	9 (18)	6 (11.1)
	With his/her partner	24 (23.1)	13 (26)	11 (20.4)
	With his/her partner and children	25 (24)	13 (26)	12 (22.2)
	With parents	3 (2.9)	2 (4)	1 (1.9)
	With another family	5 (4.8)	2 (4)	3 (5.6)
	With other people	7 (6.7)	2 (4)	5 (9.3)
	Others	3 (2.9)	1 (2)	2 (3.7)

Employment economic status	Permanent contract	35 (35.7)	18 (37.5)	17 (34)
	Temporary contract	4 (4.1)	2 (4.2)	2 (4)
	Self-employment	6 (6.1)	3 (6.3)	3 (6)
	Working without contract	8 (8.2)	6 (12.5)	2 (4)
	No work, but has a salary	32 (32.7)	13 (27.1)	19 (38)
	No work, no salary	13 (13.3)	6 (12.5)	7 (14)

Stressful event	Yes	57 (57.6)	28 (60.9)	29 (54.7)

Medication: anxiolytics	Yes	40 (37.7)	20 (40)	20 (35.7)

Hypnotics^*^	Yes	6 (5.7)	0 (0)	6 (10.7)

Alternative treatment	Yes	28 (26.4)	13 (26)	15 (26.8)

Medication: blood pressure	Yes	30 (28.3)	11 (22)	19 (33.9)

BDI	Preintervention	19.58 (5.99)	20.14 (6.32)	19.07 (5.69)

Abbreviations: SD: standard deviation.

^*^ *P* value significant (*P* = 0.018 and *P* = 0.028, resp.).

**Table 2:** Remission of depression in the overall sample.

Sample	Month	Control (*n*)	(%)	Intervention (*n*)	(%)	Difference at each follow-up		*P* value^*^
		(*n* = 56)		(*n* = 50)		(%)	(CI 95%)	
Overall	**3**	7	(12.5)	20	(40)	27.5	(11.4 to 43.6)	0.001
	**6**	11	(19.6)	21	(42)	22.4	(5.2 to 39.6)	0.012
	**9**	15	(26.8)	22	(44)	17.2	(−35.5 to 0.79)	0.064

Abbreviations: CI: coefficient interval.

^*^The difference was calculated between the intervention and control groups.

**Table 3:** Overall sample. Changes in BDI score within and between the intervention and usual care groups, with missing data replaced using the last value carried forward.

Sample	Months	Usual care group (*n* = 56)			Intervention group (*n* = 50)			Difference (95% CI) between groups (intervention group-usual care group)^**^	*P* value	SES^$^
		Mean (SD)	Difference^*^ (95% CI)	SRM^#^	Mean (SD)	Difference^*^ (95% CI)	SRM^#^	Difference		
Overall	**Preintervention**	19.07 (5.69)			20.14 (6.32)					
	**3 (postintervention)**	18.29 (6.53)	0.78 (−0.53 to 2.10)	0.16	14.68 (7.21)	5.46 (3.52 to 7.40)	0.80	−3.61 (−6.25 to −0.95)	0.008	0.55
	**6**	16.86 (6.91)	2.21 (0.19 to 4.24)	0.29	15.16 (8.92)	4.98 (2.53 to 7.43)	0.57	−1.70 (−4.75 to 1.36)	0.273	0.25
	**9**	15.66 (7.22)	3.41 (1.37 to 5.45)	0.44	14.34 (8.59)	5.80 (3.60 to 8.00)	0.75	−1.32 (−4.36 to 1.72)	0.392	0.18

Abbreviations: SD: standard deviation and CI: confidence interval.

^*^Differences were calculated between the baseline measurement and the follow-up measurement.

Positive differences indicate improvement; negative ones denote some worsening in clinical measures.

^#^SRM: standardized response mean, calculated as the mean change in score divided by the standard deviation of the change in score.

^$^SES: standardized effect size was computed as the mean difference between the intervention and control groups divided by the standard deviation of the control measurement.

A positive SRM or SES denotes improvement; a negative one denotes some worsening in clinical measures.

^**^The difference was calculated between the intervention group and the control group.

Negative differences indicate improvement in the intervention group; positive differences denote worsening in the intervention group.

Interpretation effect sizes: values of 0.2–0.5 represent small changes, 0.5–0.8 moderate changes, and >0.8 large changes.

## Abbreviations

AD: Antidepressant
CI: Confidence interval
*d*′: Cohen's effect size
IG: Intervention group
CG: Control group
ITT: Intention-to-treat
NNT: Numbers needed to be treated
PC: Primary care
PCCs: Primary care centers
PE: Psychoeducation
SES: Standardized effect size
SRM: Standardized response mean.
